# Secrets and Patents *Versus* Professional Progress

**Published:** 1898-07

**Authors:** J. Morgan Howe

**Affiliations:** New York


### International Dental Journal.

VOL. XIX. JULY, 1898. No. 7.

### Original Communications.[1]

### SECRETS AND PATENTS *VERSUS* PROFESSIONAL PROGRESS.[2]

BY J. MORGAN HOWE, M.D.S., M.D., NEW YORK.

STATEMENTS and discussions relating to secrets and to patents have been frequent enough during recent years and months to justify the conclusion that further agitation is timely, and will hasten the establishment of true moral standards.

Whether we call ourselves *a profession* or claim the connection of *a specialty* in a kindred profession, our claim to professional character, in any liberal sense, depends not on the nature or kind of work which we do as individuals, but on our relation to and treatment of each other and of the public. Quacks and charlatans work with more or less skill in the same sphere as do honorable members of the professions, but that does not give them professional standing. We began to be a professional body when less than sixty years ago our honorable predecessors organized the first dental society, issued the first dental periodical, and established the first dental college. These institutions were founded to oppose the continuance of isolation among practitioners, to break down the

[1] The editor and publishers are not responsible for the views of authors of papers published in this department, nor for any claim to novelty, or otherwise, that may be made by them. No papers will be received for this department that have appeared in any other journal published in the country.

[2] Read before The New York Institute of Stomatology.

barrier of secrecy that prevailed, and to begin the interchange and general dissemination of knowledge. It was a distinctly altruistic movement, made, despite much opposition, by the most intelligent and prosperous of our craft, who could well have afforded to allow things to remain as they were; for the benefits to accrue were more for the uninstructed and for the coming practitioners than for themselves.

There are men now living and practising who were in practice at and before that time, and I cannot do better than quote from the recently published "personal recollections" of one of them. Dr. Charles Merritt says of the absence of professional feeling among dentists, "Every new discovery or improvement made was kept a profound secret. No dentist with any reputation would allow another to see him operate, or let him get a peep into his laboratory, fearing that some of his cherished secrets would be stolen. . . . There was no organization of dentists, no real professional history. Each man was a law unto himself and pursued his own independent course, devising his own methods and practising according to his own knowledge, with no ethical code to restrain him."[1] From such a condition of isolation as this we began to emerge when the instrumentalities were set at work to accumulate a common fund of knowledge, and to establish mutual fraternal relations, obligations, and privileges, which would bind us together as a professional body.

We are accustomed to auto-congratulations in regard to the phenomenal progress that dentistry has made during recent years, but we have not always remembered the forces from which these constant advances have received continual impetus. Real professional progress in any line depends upon the general diffusion of a scientific spirit, which prompts to the imparting of information for the benefit of all, no matter how the knowledge is obtained. From knowledge thus received, added to, and in turn imparted, the successive advances are made that raise the level of intelligence of the whole professional body. By such methods the least enlightened has the means of advancement offered him as freely as any of his *confrères.* Our professional progress has been due to such liberal influences, which have prevailed, notwithstanding a constant tendency on the part of some to attribute that progress to the materials incidental to increased intelligence, instead of to educational advancement coupled with professional sentiment. A recent circular of the New York Academy of Sciences says of the investiga-

---

[1] The Dental Practitioner and Advertiser, January, 1898, pp. 12–15.

tions of scientific men, "Their results are freely given to the world for the benefit of all," and it is this characteristic and others of similar nature that must be a distinguishing mark between the professional man and the man of business who uses his knowledge in manufacture and trade.

All organizations of men necessarily require the individual to relinquish something in return for advantages and benefits received, and to accrue through like action on the part of others. If we consider even so commonplace a relation as that of citizenship, we remember that one is required to give up time to serve the State as jurors and as militiamen; to pay money in taxes; and even in some cases to give up our right to do as we wish with our material possessions. In thickly settled communities we are restricted as to how we shall locate our house on our own property, of what material we shall build it, and what the character of the conveniences shall be that we put inside it. All such and many other instances of yielding up of rights and privileges, for which the barbarian would fight, are peacefully conceded for the general good, as civilization advances. And similarly, as the processes of evolution proceed and society becomes more complex, other organized bodies must require concessions peculiar to themselves, which also imply the giving up of rights or possessions in return for benefits conferred. There is no doubt about the legal right of an individual to withhold information regarding any knowledge he may acquire; but if he belongs to a professional body, and the information he retains for his own use relates to the work of his *confrères*, he is evidently returning to the practice of those who thus withheld knowledge before the organization of the professional body began. Relevant to the provision of the medical code against the use of nostrums, an editorial in the *Dental Cosmos* not much over two years ago[1] objected in the strongest terms to the advertising of nostrums in medical and dental journals, as well as to the use of such compounds by practitioners, but all the force of the article was directed against medicinal preparations. *Nostrum* is the Latin word for *our*, *ours*, and is supposed to be applied, as it is, from "the habit of quacks and other advertisers of claiming special virtue for their wares as 'our own make,'" and, although more commonly applied to medicines, is equally applicable "to any scheme or device of a quack or charlatan."[2] I submit that no use of the word could

---

[1] Dental Cosmos, vol. xxxvii. p. 723.

[2] Century Dictionary.

be more appropriate than its application to the secret formulæ of alloys and cements that are offered us in all dental journals. Dr. Ottolengui has also given a valuable contribution on "secret remedies,"[1] but he also confined his attention to drug compounds, and ignored all consideration of the compound remedies for dental caries that we are all using constantly. Whether it has occurred to either of these gentlemen, who edit journals for supply houses, that the secret and nostrum character of the alloys and cements, in which their firms and most dealers are interested, is the same as that to which they object in drug compounds, does not appear; but I am sure the logic of their convincing arguments against secret remedies and nostrums will lead most of us to include these filling compounds as differing not at all in principle from other secret preparations.

During the last three years we have had very full and valuable publications from Dr. Black, as the result of his scientific study of amalgams, but as yet it seems as if we were receiving no benefit from the information he has imparted, as every manufacturer and dealer in supplies has his own compound, which he is most anxious to sell.

Dr. Crouse, for the Dental Protective Supply Company, while acknowledging his indebtedness to Dr. Black's publications and suggestions, by adding to these his own investigations, has "solved the problem at last" and produced "the only perfect alloy," but he objects to revealing the results of his studies or the composition of his product. I question the propriety of calling this compound "Fellowship" alloy, because it embodies such a flagrant violation of that professional sentiment which expects members of the body to contribute of their knowledge to the common fund as freely as they have received. Dr. Crouse, in reply to a letter written him in January last, calls the proposition to publish the formulæ of alloys "absurd,"[2] and thinks that such publication has been "detrimental to the dental profession," and that it has been a reason for the production of "worthless preparations" and of constant variations in quality. He thinks he can "best help my (his) profession" "not by giving them a formula," especially in view of the cost to the Supply Company of a metallurgist, months of time, and expensive instruments. He says, "Why should the conclusions arrived at be made public?" "This course would be manifestly unjust to the stockholders."

---

[1] Items of Interest, April, 1898.

[2] Dental Digest, February, 1898.

The statement is from the manufacturers' stand-point. His interest in maintaining secrecy is frankly admitted, while, on the other hand, he claims it is good for the "profession" to be kept in ignorance, because the "worthless preparations" on the market are the result of the publication of formulæ.

It does not seem as if this reasoning is convincing or acceptable to dentists who regard their vocation as a professional one. We are all very thankful, I am sure, to Dr. Crouse for his services in connection with the Protective Association, but we may decline, I hope without offence, the proffer of a guardianship that would shield us from too much knowledge. On the contrary, it is time in this stage of our maturity to demand such knowledge, even if stockholders have to accept less dividends.

Dr. Flagg and a very few other manufacturers of alloys have published their formulæ. Dr. Keller published not only the composition of alloys that he made, but also the results of analyses of sixty-five secret compounds then upon the market.[1] There would seem to be no objection possible to the preparation and sale of known compounds by a professional man to his *confrères*. Painstaking care and skill in such work—as in everything—is worth paying for; but such a question by a dentist as "Why should the conclusions arrived at be made public?" takes us back to early days. It is the same question in effect that those men asked who opposed the efforts of Hayden, Harris, Parmly, Flagg, and others, who began the agitation for the establishment of our professional relations. It has a tone of antiquity about it that in these days of societies and journals sounds strangely coming from a member of a profession. Although paradoxical, it shows that we are a youthful body. Our willingness to continue buying and using secret compounds, for which extravagant claims are made, must be due to mental qualities similar to those which keep up the sales of quack medicines to the general public. But those who claim to be professional men should certainly rise above such a level. We cannot blame the proprietors for taking advantage, as business men, of the desire to buy what they have for sale; but to argue that it is not good for professional men to know what they are using is certainly unique. Of course it is recognized as an easy matter to analyze any inorganic compound, so that the composition may certainly be known, if it is really desired; but the question of interest just now is, What relation does a so-called professional man hold to his fellows

---

[1] American System of Dentistry, vol. iii. p. 813.

who withholds information for gain? I contend there is as much justification for expecting and demanding the giving up of secrets as there is that members of a professional vocation should conform in any other way to the consensus of opinion concerning what is favorable to progress. There is no more right or reason in an interdiction against public advertising than against making, selling, or holding an interest in a secret nostrum, for both are an injury to the profession as such. The moral status of any course of action may be justly estimated by calculating the effects of such conduct on the whole body, if all its members resorted to similar behavior. Applying such a test to secrecy, the conclusion must be, if it would be an injury to the professional body for all our members to withhold information, to reserve for their personal benefit everything they can turn to their own advantage, and cease all efforts to enlighten for the sake of elevating professional status; then there is no question that he who claims exemption from the liberal usage—which brought us out of the chaotic condition of former days—is working an injury to the extent of his influence.

Solicitude has been recently expressed in regard to the increase and success of the so-called "dental parlors," conducted with brazen advertising of "painless" operation and flaunting college degrees. Why should these men be asked to give up their legal right to use such means as they find of pecuniary advantage. They have as good reason for claiming their right to do as they please in their method of obtaining business as have those who in any other way seek their own .interests, without regard to their effects upon the profession of which they claim to be members. Their methods are forbidden because they injure the professional body. The right must be relinquished for the general good. A general desire among dentists to know the composition of all the compounds they use, and a determination to deliver themselves from their present bondage, together with the establishment of an antisecret ethical standard by dental societies and schools, will do more than anything else to make publications, such as Dr. Black's, of service to all dentists, and at the same time make all dentists more intelligent and better professional men. It is an entirely practical proposition that we should have compound filling-materials made from known formulæ by experts, who will serve us as willingly as pharmacists now serve physicians; and the practical way to bring about the change is for dentists to cease using secret compounds. Publication of formulæ will be general when it is found to pay. Chemists and metallurgists will fill orders when they are employed.

It would be superfluous to take time here in condemning medicinal nostrums; the arguments against them, before mentioned, are conclusive; but there is no consistency in seeking to maintain a professional standard in regard to one set of materials and permitting, without protest, the level of trade to be continued regarding other substances, in which we are fully as much interested.

The only logical position we can occupy is that full and free dissemination of information concerning all things connected with our work is required for professional development, and no less will be acceptable.

If there is anything more inconsistent with professional progress than secrecy it is the revelation of ideas by means of letters patent. There is no doubt that the intention of patent laws is to stimulate invention, and it will not be denied that they have done so to some extent. But it is well known that a decided objection exists in the medical profession against the obtaining of a patent by a physician on any invention pertaining to his professional vocation. Among dentists such objection has not been so generally recognized, and there is probably some difference of opinion among those who have no direct pecuniary interest in fostering the belief that patents are a benefit and not an injury to the true interests of our specialty. On this point I desire to discuss the matter further, and to make some application to present conditions.

There is probably nothing under human control that is either wholly good or totally evil. This applies to men's characters and to all their schemes and devices. I do not propose to argue that the effects of patents are entirely evil. It is claimed and may be true that patent laws have been of great benefit to the public by stimulating and rewarding invention, but if so, the public has, nevertheless, been obliged to pay very dearly for the advantage. Time would fail to discuss the subject on this broad plane, but I wish to present reasons for the claim that a profession cannot permit its members to patent inventions, because the evil effects far exceed any possible good. Medical and other scientific men are stimulated to devise, invent, or discover, so far as in them lies, by a scientific or professional spirit; and the commercial instincts of some are, no doubt, restrained by the code of ethics which forbids them from patenting productions that may be of use to their *confrères*. They may copyright their books or other productions, and they receive pay for all forms of teaching, as do all professional men; so the objections to their obtaining a patent on devices for technical use cannot be on the score of their remuneration increasing the cost of the article. Dentists who have argued for the privilege of patenting, while remaining in good standing, have claimed that it is no more than is conceded to the man who copyrights his book; but it is more and different; because the man who obtains remuneration through copyright does no harm, but the professional man who seeks reward by means of patent does harm to the body of which he is a member, through power which he holds, or conveys to others, to repress the productions of his *confrères*.

Mr. David McGregor Means, of the New York bar, wrote on the difference between the general effects of copyright and patent right at the time of the discussion of international copyright.[1] He said, "This right, far from being mysterious and questionable, is one of the simplest and clearest with which jurisprudence can deal, and is, moreover, exactly defined by the term in common use,—'copyright.' It is the right to copy, to imitate, to consciously repeat what has already been done, and negatively the idea of originality in labor, in thought, or in discovery. The expression 'property in ideas' is a peculiarly unfortunate one to apply to this right. It implies either that the holder of copyright and his assigns are the only persons entitled to think certain thoughts, to entertain certain ideas, or that they alone may practically apply the thoughts or make use of the ideas. Neither of these implications has the slightest support in the facts of the case."

But "this expression is not improperly applied to the right conferred by letters patent, which is of an entirely different character from copyright. The holder of a patent cannot, it is true, prevent others from thinking his thoughts and having his ideas, but he can prevent them from making any practical use of these thoughts and ideas. He can prevent them from moving portions of matter in a particular manner,—that is, in conformity to certain ideas; and as all labor consists in moving matter, he has a legal monopoly of certain modes of labor. There may have been a hundred men working independently, to each of whom the same idea would have occurred at almost the same time, but the one that patents it may forbid the rest of mankind from applying it in the use of their property. The idea may have come to the discoverer by accident and without labor on his part, while accident has prevented those who sought for it from finding it; that makes no difference with his legal right." Copyright "confers no such monopoly. The ideas contained in a copyrighted book are no one's

---

[1] "Concio ad Clerum," the Nation, July 10, 1890.

property. They may be, every one, expressed in other words which may be printed and published; they may be memorized; they may be applied to any use by any person, and the right of the author is not infringed. By this right no human being is prevented from making any use of his mind or of his property that he pleases, while it may suggest to him novel uses of both. By it no man is hindered from doing what he would have done did it not exist." It is clear from these statements that there is not only a radical difference between copyright and patent, but that the latter must necessarily be a hinderance to professional progress in material devices, as well as in the diffusion of professional feeling.

We know from experience and observation in our own vocation that patents have been a hinderance to progress by preventing improvements being made on devices which could be made more practical; by preventing the production of new devices if they have in them even a modicum of the ideas that have been patented; and by suppressing the production of other patented inventions by shelving, when the sale of something in the market would possibly be reduced by the substitution of an improved device.

There are many inventions now hidden from general knowledge that would facilitate work and suggest further advances, but for the pecuniary interest created by patents in smothering such devices in order that the market value of certain other things may not be lowered.

All these repressive powers are exercised by business men to whom dental patents are sold. Besides, there is the power to harass, annoy, and oppress by the license requirements of patent-holders, of which we had an extended knowledge under the Vulcanite Company, and more recently a foretaste of similar bitter experiences with the Tooth Crown Company. These organizations were based on patents obtained by dentists, and sold to those who organized the corporations for the purpose of exacting oppressive tribute from the dentists of the whole country. Much credit is due to Dr. Crouse for organizing the opposition to the latter company; but resistance has been successful only in the degree that the patents have been proved invalid. If the patents of the Tooth Crown Company had been held valid, the dentists of the country would now be in the grasp of that company. We have escaped thus far only by virtue of the invalidity of their patents. When another company is organized with a basis of valid patents, we will not escape its exactions, although the ablest legal talent and every dentist in the land contribute to the defence. Dentists have only to go on patenting whatever they can, and selling their patents to whoever will buy, and the organization of such another company will not be long deferred. If the patents prove valid, escape is impossible.

Then there is also the additional obstacle to professional growth in the patenting of modes, or methods, of performing operations. Under such patent powers a man may be punished for following the dictates of his judgment, in doing what his patient needs to cure his disease, correct his deformity, or even save his life.

In all these ways patents are an injury to any organized body of a professional character, and no member of such a body has a moral right to inflict such an evil on the body from which he has drawn that which has helped to make him what he is. I have not mentioned the increased price for patented things as detrimental to professional progress, because relatively it is of minor consequence. Objections to patents in a profession are not at all based on a desire to evade remuneration to the inventor through a reasonable increase of price, but on the fact that he cannot secure remuneration by means of the patent without inflicting serious injury on the profession. Ready acquiescence in copyright shows plainly that no professional man objects to compensating his *confrère*, but the power to monopolize ideas, to forbid improvements, to shelve new inventions, to impose humiliating as well as exorbitant license terms on patented methods as well as things, in addition to remuneration, deservedly excites opposition. Such power is opposed to the fundamental ideas that distinguish a profession from a trade; and the more certainly valid the patent proves to be, the more resistless the power to check professional progress.

The justice of the plea favoring patents because the inventor ought to be paid is called in question by the fact, well known to most of us, that original inventors really get very little when they patent their inventions. When money is made from patents, it is almost always the business men, who buy and exploit them, that get it. And then original inventors among us are not so eager to patent their contrivances as are those who appropriate their ideas. Many of our most valuable devices have been freely given, and many patents have been obtained long afterwards on things that were originally given to their *confrères* by men of true scientific spirit. The screw for use in operative dentistry was patented years after it had been used and described by Dr. Dwinelle. Variations in the form of the matrix have been patented long after the original idea was given us by Dr. Jack; and even when a patent is not possible, the same mercenary spirit shows itself in appropriating the inventions of others without credit, as in the case of Dr. I. F. Wardwell's invention twenty years ago of what are now called "detachable porcelains." Although described and illustrated in two text-books, they are now being advertised and sold as the product of another's mind.

Dr. Crouse is authority for the statement[1] that ninety per cent. of all patents on our appliances have been invalid; and it would seem that this must be in great measure due to the penchant for appropriation—conscious and unconscious—that has obtained such sway among dentists.

In addition to this, the statement is made[2] that all patents on mechanical processes, such as are used in our operations, have been declared invalid by the United States Supreme Court. So there remains less than ten per cent. of all our patents that have the questionable virtue of validity, and for the alleged benefits of this modicum we are actively keeping up a system against which at the same time we must protect ourselves by a continuous and desperate series of legal battles.

Among physicians and other scientific men the patent is not a necessary stimulus, because it has been, and is constantly being, shown that they are willing and even anxious to contribute their ideas in tangible, as well as in other forms, without seeking other reward than that which comes with recognition of ability.

One has only to visit the establishment of any large surgical or scientific instrument-maker to see a great variety of complicated as well as simple instruments for all medical or scientific purposes not protected by patent, and these are constantly being modified for improvement and increased in number. Such an exhibit shows that there is no lack of willingness to devise without the stimulus of patents; and it is evident, also, that the manufacturers of these devices are willing to make them, although not protected against the competition of other makers.

Denunciation of manufacturers and license corporations, patent-holders, because of alleged extortion in the price of patent permits or articles, appears to be inconsistent and ill-directed so long as dentists themselves are keeping up the supply of patents, which they sell generally for a paltry sum to those who really are the money-makers in the matter. The business men who make and

---

[1] Dental Digest, February, 1898.

[2] Ibid., March, 1898.

sell our supplies, and buy patents when they think them valuable, either to use or to prevent use, are merely taking advantage of the business opportunity which is furnished them by members of our own body. They are not professional men and ought not to be judged by professional standards; but for ourselves such ethical rules should be established as will disqualify from good professional standing those who obtain and sell the power to repress and harass their colleagues, for there is every prospect of the evils of patents, both valid and voidable, being in the future a greater curse than they have been already. We have the matter in our own hands as a professional body. If our societies take the matter into consideration and establish the best code of ethics attainable in our present status of professional development, if the students in our colleges are surrounded by a scientific atmosphere that scorns secrecy and repression, and if such a liberal professional sentiment is diffused as to make dentists generally interested in the composition of their materials, and to cause them to regard their professional standing and the interests of the professional body of more value than the sum they may hope to get from a patent, a great step forward will be made in professional progress. The effort being made by Dr. Ottolengui for a general petition to Congress for an amendment to the patent law, so as to prevent the issuing of patents on operations and processes, ought, if feasible, to receive the hearty approval and co-operation of every one of us as an effort to lessen the evils of patents. I would approve of all and every means that can be made effectual to prevent or diminish the patent mischief at its fountain-head. The Dental Protective Association, under the able direction of Dr. Crouse, the dentist, has delivered us from the exactions thus far of those whose patents have proved voidable, but neither it nor any other defender can deliver us, as before remarked, from the exactions of valid patents.

We certainly need a protective association to defend us against the demands of those who have no just claims, and no such association could be better managed than the one now in existence, I am sure. But we need also to be delivered from the evils of valid patents, and there seems to be no other way than to raise the level of our morals sufficiently to reduce the number of those to a minimum who are willing to injure the professional body for the hope of gain.

We have undoubtedly been advantaged through the enterprise of manufacturers, but we have also been injured through their influence. There can be no doubt that trade interests are served by the systems of secret formulæ and patents. Dentists must determine through their proper organizations in which direction they will move,—towards or away from the standard of a liberal profession?

The developed policy of the Dental Protective Supply Company, under the management of Dr. Crouse, the manufacturer, in conducting the business strictly on business lines, without regard to the effects of secrets and patents on professional progress, is likely to confuse the moral sense, because Dr. Crouse is also a dentist of prominence and distinction, and because other dentists are shareholders in the business, and still more dentists are being solicited to become shareholders. Good dividends are calculated to cause one to consider the methods or results of obtaining them, with a bias; and we may be sure that these professional shareholders in dental trade will hardly escape such influence. This is only one more deteriorating influence added to those that have been longer in operation.

Professor Peirce, of the Pennsylvania College of Dental Surgery, in a very recent paper,[1] makes a lament—in which I can but join—over the degrading influences of the commercial spirit pervading educational institutions and the inconsistency—or worse—of examining boards. I would suggest that the desired upward drift could as effectually begin in educational institutions as elsewhere. Let teachers begin to inculcate, by example and by precept, the lesson that the moral status of a profession determines its position in public estimation, and in great degree that also of every individual member, and the ascent will begin.

But if teachers and leaders grasp at patent prizes, how can the rank and file be expected to forego any chance for expected gain that seems within their reach? If colleges countenance and use secret compounds of certain kinds, what weight can attach to the professors' denunciation of nostrums of another kind. If students and practitioners imbibe—from teachers and journals and manufacturers—the idea that professional progress consists in patent devices, mechanical facilities, and secret compounds, what logical objection can be made to the embodiment of this notion in "dental parlors," in department stores, or elsewhere?

All professions are liable to have both their character and reputation impaired by the degrading influence of members who are indifferent or ill-informed regarding moral considerations; but dentistry

---

[1] International Dental Journal, April, 1898, p. 207.

is probably the most vulnerable specialty in this respect, because it has not yet acquired the prestige and reputation of the older bodies, nor has it so far freed itself from those unprofessional ideas and practices that are connected with its embryonic state. We can the less afford, therefore, to ignore the need of ethical development, or to pass unnoticed the influence of practices that would cause retrogression.

These presentments are made in the hope of exciting interest and discussion, so that we may be brought nearer to the acceptance of the truth. If there are interests that "put golden padlocks on truth's lips," it behooves us to recognize the fact.

